# The efficacy and safety of acupuncture in women with primary dysmenorrhea: A systematic review and meta-analysis

**DOI:** 10.1097/MD.0000000000011007

**Published:** 2018-06-18

**Authors:** Hye Lin Woo, Hae Ri Ji, Yeon Kyoung Pak, Hojung Lee, Su Jeong Heo, Jin Moo Lee, Kyoung Sun Park

**Affiliations:** a Department of Korean Medicine Obstetrics and Gynecology, Kyung Hee University Hospital at Gangdong; b Department of Korean Medicine Obstetrics and Gynecology, College of Korean Medicine, Kyung Hee University, Seoul, Republic of Korea; c Masters of Sciences in Oriental Medicine, Dongguk University in Los Angeles, CA; d Dongbu Central Hospital; e Department of Clinical Korean Medicine, Graduate School, Kyung Hee University, Seoul, Republic of Korea.

**Keywords:** acupuncture, dysmenorrhea, meta-analysis, primary dysmenorrhea, systematic review

## Abstract

**Background:**

This systematic review aimed to evaluate the current evidence regarding the efficacy and safety of acupuncture on primary dysmenorrhea.

**Methods:**

Ten electronic databases were searched for relevant articles published before December 2017. This study included randomized controlled trials (RCTs) of women with primary dysmenorrhea; these RCTs compared acupuncture to no treatment, placebo, or medications, and measured menstrual pain intensity and its associated symptoms. Three independent reviewers participated in data extraction and assessment. The risk of bias in each article was assessed, and a meta-analysis was conducted according to the types of acupuncture. The results were expressed as mean difference (MD) or standardized mean difference (SMD) with 95% confidence intervals (CIs).

**Results:**

This review included 60 RCTs; the meta-analysis included 49 RCTs. Most studies showed a low or unclear risk of bias. We found that compared to no treatment, manual acupuncture (MA) (SMD = −1.59, 95% CI [−2.12, −1.06]) and electro-acupuncture (EA) was more effective at reducing menstrual pain, and compared to nonsteroidal anti-inflammatory drugs (NSAIDs), MA (SMD = −0.63, 95% CI [−0.88, −0.37]) and warm acupuncture (WA) (SMD = −1.12, 95% CI [−1.81, −0.43]) were more effective at reducing menstrual pain. Some studies showed that the efficacy of acupuncture was maintained after a short-term follow-up.

**Conclusion:**

The results of this study suggest that acupuncture might reduce menstrual pain and associated symptoms more effectively compared to no treatment or NSAIDs, and the efficacy could be maintained during a short-term follow-up period. Despite limitations due to the low quality and methodological restrictions of the included studies, acupuncture might be used as an effective and safe treatment for females with primary dysmenorrhea.

## Abstract 2

Supplemental Digital Content is available in the text

## 1 Introduction

Primary dysmenorrhea is defined as cramping pain during menstruation without any identifiable pelvic pathology,^[1]^ and it affects most women throughout the menstrual years.^[2]^ Many studies have reported that the prevalence of primary dysmenorrhea varied from approximately 50% to 90%,^[3–6]^ and 13% to 51% had to limit daily activities, such as school or work absenteeism.^[2]^ In the consensus guidelines of primary dysmenorrhea,^[7]^ nonsteroidal anti-inflammatory drugs (NSAIDs) and oral contraceptives (OCs) are recommended as first-line treatments. However, some patients did not experience pain reduction with NSAIDs and did experience side effects such as nausea, dyspepsia, headache, or drowsiness.^[8,9]^ In addition, OCs may not be suitable for patients attempting to become pregnant, and might cause adverse effects such as nausea, vomiting, weight gain, or vaginal bleeding.^[10,11]^

Acupuncture, derived from China, is a therapeutic modality using the insertion of fine needles with the concepts of *Yin* and *Yang* and the circulation of *qi*. Acupuncture acts primarily by stimulating the nervous system, by local effects due to local antidromic axon reflexes, and by releasing opioid peptides and serotonin. Today, acupuncture is regarded as part of conventional medicine. It is no longer only “alternative medicine,” and it is used in Western medicine.^[12]^ In particular, acupuncture has been widely used to alleviate diverse pains^[13]^ including menstrual pain.

Many clinical trials had been conducted to show efficacy of acupuncture on menstrual pain, and 6 systematic reviews (SRs) have been previously conducted to evaluate the efficacy of acupuncture on primary dysmenorrhea.^[14–19]^ However, the previous SRs included acupressure, the stimulation of acupoints without skin penetration,^[14–18]^ which made the evaluation of acupuncture difficult. Some studies analyzed all types of acupuncture together,^[14–17]^ which increased the heterogeneity. One latest study^[19]^ included all the types of acupuncture except acupressure and analyzed the results separately, but it did not include newly published studies in 2017. Thus, we found it necessary to conduct a study with rigorous criteria that excluded acupressure and included all other types of acupuncture that penetrate the skin, such as embedding therapy, and to synthesize the data according to the type of acupuncture to reduce heterogeneity. We conducted this study with these criteria to determine the efficacy and safety of acupuncture on primary dysmenorrhea.

## 2 Methods

### 2.1 Study registration

The protocol for this study was registered in PROSPERO: CRD42017069258.

### 2.2 Eligibility criteria

#### 2.2.1 Types of studies

We included all randomized controlled trials (RCTs) that measured pain intensity and related outcomes to evaluate the efficacy of acupuncture in women with primary dysmenorrhea. Case studies, case series, noncontrolled trials, review articles, letters, conference papers, abstracts, and poster presentations were excluded. Studies not written in English, Chinese, or Korean were also excluded.

#### 2.2.2 Types of participants

We included female patients of reproductive age suffering from primary dysmenorrhea. The definition of primary dysmenorrhea was based on cyclic pelvic pain during menstruation without any gynecological pathology such as endometriosis, adenomyosis, or uterine myoma. Patients with secondary dysmenorrhea or serious medical conditions were excluded.

#### 2.2.3 Types of interventions

Manual acupuncture (MA), electroacupuncture (EA), auricular acupuncture (AA), and any other type of acupuncture using needle insertion were included in our study. Pharmacopuncture and acupressure were excluded. Other types of acupuncture that are rarely used in Korean clinical practice, such as eye acupuncture and floating acupuncture were also excluded. Types of control interventions included in our studies were no treatment, placebo acupuncture, and oral medications such as NSAIDs and OCs. Herbal medicines or other traditional medicine treatments used in the control group were excluded from our study.

#### 2.2.4 Outcomes

The primary outcome was pain intensity after the intervention period as measured by any validated scale, such as the visual analog scale (VAS) or numeric rating score (NRS). The secondary outcomes were pain relief measured by total effective rate (TER) or improvement rate; related symptoms measured by the seven-point verbal rating scale (VRS), Cox menstrual symptom scale (CMSS), Cox retrospective symptom scale (RSS), or menstrual symptom score (MSS); quality of life as measured by the 36-item Short Form health survey (SF-36); pain intensity after a follow-up period; and adverse events (AEs).

### 2.3 Data sources

The following databases were searched for articles published from the database's inception to December 2017: MEDLINE, Embase, Cochrane Central Register of Controlled Trials (CENTRAL, The Cochrane Library), Allied and Complementary Medicine Database (AMED), Citation Information by NII (CiNii), China National Knowledge Infrastructure (CNKI), Chinese Science and Technology Periodical Database (VIP), Wanfang, Oriental Medicine Advanced Searching Integrated System (OASIS), and the Korean Traditional Knowledge Portal (Korean TK). There was no language restriction. We used Medical Subject Heading (MeSH) terms and their synonyms, and modified the terms according to the strategy of each database. The search terms used are shown in Supplemental Search Terms List.

### 2.4 Study selection

All studies found based on the search results were saved into EndNote; duplicated studies were excluded. After deleting the duplicates, 3 reviewers, WHL, HSJ, and LHJ, selected the relevant studies independently by title and abstract, and finally selected the included studies using the full text. Any disagreements were resolved by discussion among the 3 reviewers and an arbiter, PKS.

### 2.5 Data extraction

Three authors, WHL, HSJ, and LHJ, extracted data from the included studies according to the predetermined data forms. The following items were extracted: baseline demographics (journal, author, and year of publication); participants (sample size, sex, and age); intervention (type of acupuncture, periods, and frequency of treatment, and follow-up period); control; and outcome.

### 2.6 Risk of bias assessment

WHL, HSJ, and LHJ independently assessed the risk of bias for each included study using the following criteria from the *Cochrane Handbook for Systematic Reviews of Interventions*^[20]^: random sequence generation; allocation concealment; blinding of participants and personnel; blinding of outcome assessment; incomplete outcome data; and selective reporting. We assessed these 6 criteria using “Low” (“L”), “Unclear” (“U”), and “High” (“H”) as a key for judgements. “Low” indicated a low risk of bias, “Unclear” indicated that the risk of bias was uncertain, and “High” indicated a high risk of bias. Disagreements were resolved by discussion among the 3 reviewers and an arbiter, Park KS.

### 2.7 Data synthesis

In our review, for studies using the same type of acupuncture, comparator, and outcome measures, the meta-analysis was performed using Review Manager software (RevMan v. 5.3). To assess the effect of acupuncture on primary dysmenorrhea, dichotomous data were analyzed using a risk ratio (RR) with 95% confidence intervals (CIs), and continuous data were analyzed using mean differences (MD) and 95% CIs or standardized mean differences (SMD) with 95% CIs if different scales were used. The chi-square and *I*^2^ tests were used to assess statistical heterogeneity.^[20]^ If *I*^2^ > 50% or *P* < .1, we considered that there was substantial heterogeneity among the trials, and if *I*^2^ > 75%, we considered that there was serious heterogeneity. When serious heterogeneity was indicated, we found sources of heterogeneity by subgroup or sensitivity analysis. Subgroup analysis was conducted according to the treatment periods, and sensitivity analysis was done by excluding each heterogeneous trial. In case of substantial heterogeneity, a random effects model was used; otherwise, a fixed effects model was used to synthesize the data. However, if there were few studies for pooling, a fixed effects model was implemented because it is difficult to obtain a precise estimate of the between-studies variance.^[21]^ If the number of the appropriate studies was only 1, or data were unsuitable for quantitative synthesis, descriptive synthesis of the findings was performed. If the number of studies for pooling was more than 10, publication bias was assessed using a funnel plot.^[22]^

## 3 Results

### 3.1 Study selection

**Figure 1:**
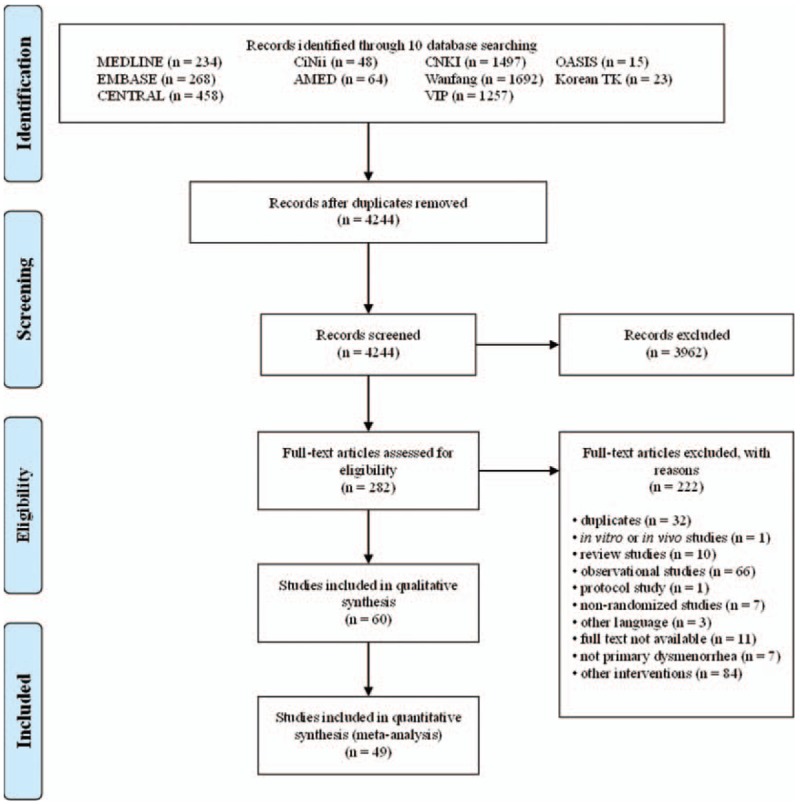
PRISMA flow chart of the study selection process used for meta-analysis. PRISMA = Preferred Reporting Items for Systematic reviews and Meta-Analyses.

A total of 4244 articles were screened, and 3962 were retrieved. The full texts of 282 studies were reviewed; 222 did not meet our inclusion criteria. Finally, 60 RCTs meeting our criteria were included. All studies were published between January 1987^[23]^ and November 2017.^[24]^ Forty-four studies were published in Chinese,^[24–67]^ 15 in English,^[11,23,68–80]^ and 1 in Korean.^[81]^Figure 1 shows a Preferred Reporting Items for Systematic reviews and Meta-Analyses (PRISMA) flow of the study selection process.^[82]^

### 3.2 Study characteristics

#### 3.2.1 Patients

A total of 3171 patients were treated with MA, EA, warm acupuncture (WA), AA, or catgut embedding therapy (CET); 2730 control patients received no treatment, placebo acupuncture, or oral medications. Of 60 trials, 55 were conducted in China (5653 patients),^[24–71,73–79]^ and 1 each was conducted in America (22 patients),^[23]^ Turkey (35 patients),^[72]^ Australia (92 patients),^[80]^ Thailand (52 patients),^[11]^ and South Korea (47 patients),^[81]^ respectively. The age range of the participants was 10 to 43 years. Table 1  summarizes the characteristics of the included studies.

**Table 1:**
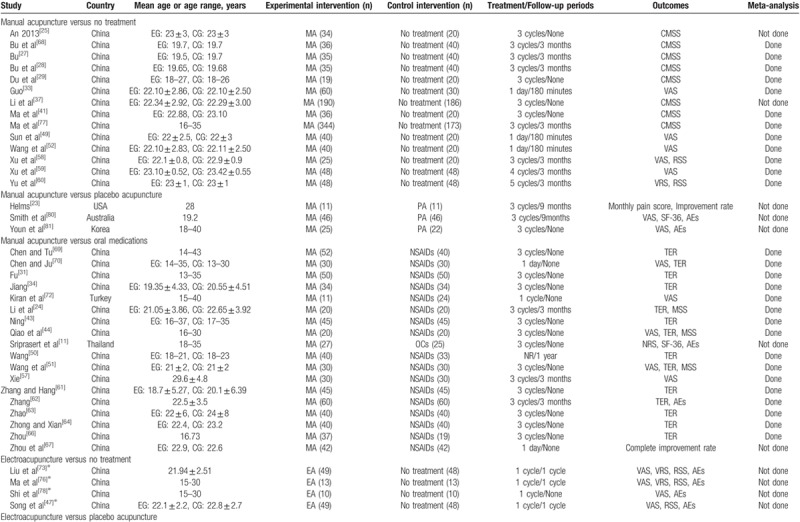
Summary of the included studies.

**Table 1 (Continued):**
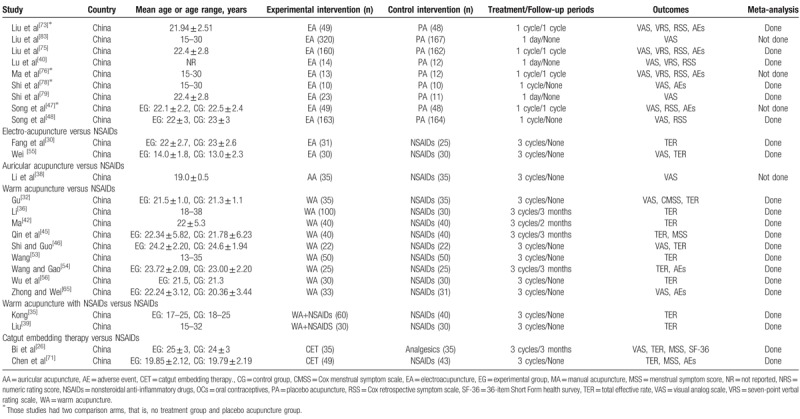
Summary of the included studies.

#### 3.2.2 Acupuncture interventions

Of 60 trials, 35 used MA,^[11,23–25,27–29,31,33,34,37,41,43,44,49–52,57–64,66–70,72,77,80,81]^ 11 used EA,^[30,40,47,48,55,73–76,78,79]^ 11 used WA,^[32,35,36,39,42,45,46,53,54,56,65]^ 1 used AA,^[38]^ and 2 used CET.^[26,71]^ The number of acupoints used varied from 1 to 21. The most frequently used point was *Sanyinjiao* (SP6), followed by *Guanyuan* (CV4), *Diji* (SP8), *Cialiao* (BL32), *Zusanli* (ST36), *Xuehai* (SP10), *Taichong* (LR3), *Zhongji* (CV3), *Shiqizhui* (EX-B8), and *Shenshu* (BL23). The only point used in 9 trials that used EA was *Sanyinjiao* (SP6). Twenty-one trials used different acupoints or added acupoints based on traditional Chinese medicine patterns.^[26,31,35,36,50,55,57–60,62–64,66,70,71,80,81]^ Treatment duration ranged from one day to 3 menstrual cycles; 25 trials included follow-ups,^[23,24,26–28,33,36,42,45,47,49,50,52,54,57–60,62,68,73,75–77,80]^ which varied from 180 minutes to one year. The time of intervention started before menstruation started in 31 trials,^[27–32,34–38,42–45,51,53–60,62,63,65,66,68,69,72]^ when menstruation started in 4 trials,^[46,73,75,78]^ when pain occurred in 10 trials,^[24,33,40,41,47–49,52,74,79]^ and continuous treatment except for menstrual periods in 6 trials.^[11,23,26,39,71,80]^*De-qi* sensation was performed in most trials, but 4 studies did not mention about *De-qi* sensation.^[23,38,41,50]^ Additional interventions to acupuncture were included in 15 trials.^[11,35,39,40,47,48,59,60,73–76,78–80]^Table 2    shows the acupuncture points and treatment methods of the included studies based on STandards for Reporting Interventions in Clinical Trials of Acupuncture (STRICTA) recommendations.^[84]^

**Table 2:**
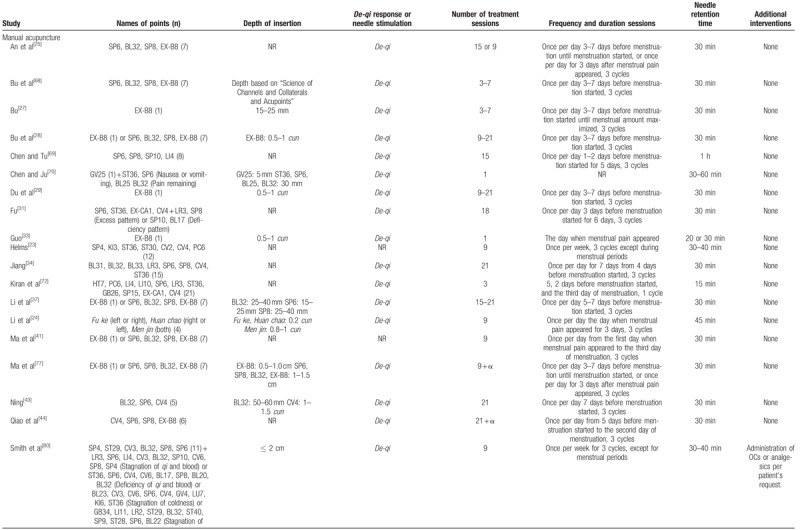
Acupuncture interventions of the included studies based on STRICTA recommendations.

**Table 2 (Continued):**
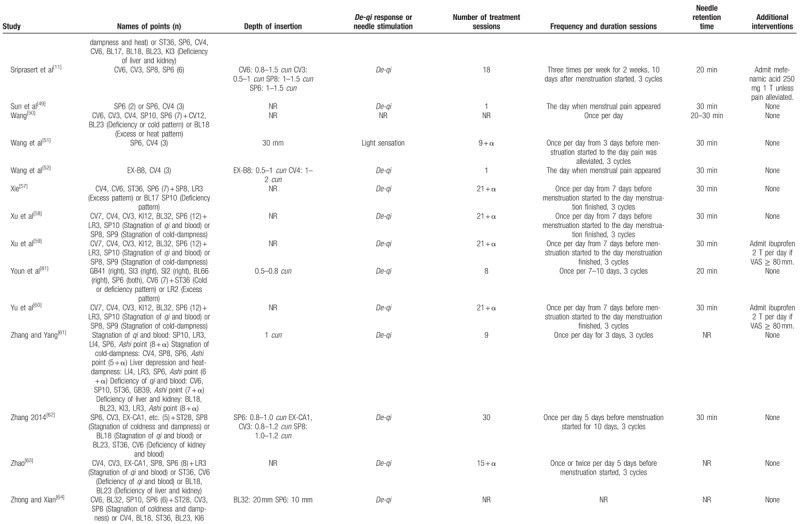
Acupuncture interventions of the included studies based on STRICTA recommendations.

**Table 2 (Continued):**
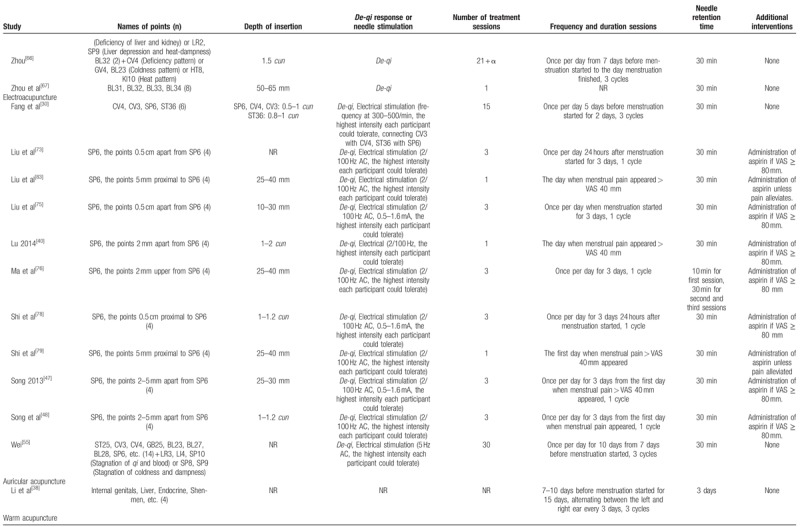
Acupuncture interventions of the included studies based on STRICTA recommendations.

**Table 2 (Continued):**
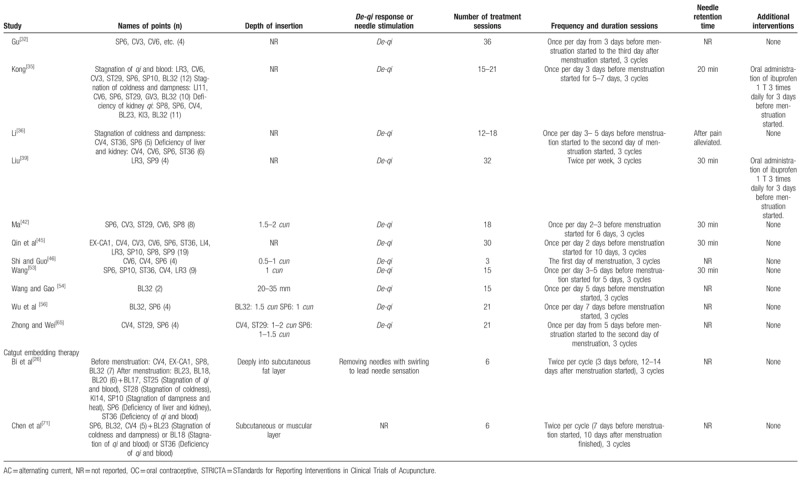
Acupuncture interventions of the included studies based on STRICTA recommendations.

#### 3.2.3 Control interventions

Of the 35 trials that used MA, 14 compared MA to no treatment,^[25,27–29,33,37,41,49,52,58–60,68,77]^ 3 compared MA to placebo acupuncture,^[23,80,81]^ and 18 compared MA to oral medications.^[11,24,31,34,43,44,50,51,57,61–64,66,67,69,70,72]^ Most of the medications were NSAIDs, and only 1 was an OC.^[11]^ Of the 11 trials that used EA, 4 compared EA to nonacupoint EA (placebo EA), or no treatment,^[47,73,76,78]^ 5 compared EA to nonacupoint EA,^[40,48,74,75,79]^ and 2 compared EA to NSAIDs.^[30,55]^ Of 11 trials that used WA,^[32,35,36,39,42,45,46,53,54,56,65]^ 2 trials compared WA plus NSAIDs to NSAIDs^[35,39]^ and 9 trials compared WA to NSAIDs.^[32,35,36,39,42,45,46,53,54,56,65]^ One trial compared AA to NSAIDs.^[38]^ Two trials compared CET to NSAIDs.^[26,71]^ All of the placebo controls used nonacupoint acupuncture, not sham acupuncture.

#### 3.2.4 Outcome measures

Twenty-seven trials measured pain intensity using VAS,^[26,32,33,38–40,44,46–49,51,52,55,57–59,65,70,72–76,78,79,81]^ 1 used NRS,^[11]^ 9 used CMSS,^[25,27–29,32,37,41,68,77]^ 5 used VRS,^[40,60,73,75,76]^ and 8 used RSS.^[40,47,48,58,60,73,75,76]^ Thirty trials measured pain relief.^[23,24,26,30–32,34–36,39,42–46,50,51,53–56,61–64,66,67,69–71]^ Six trials measured overall menstruation symptoms using MSS.^[24,26,44,45,51,71]^ Three trials measured the quality of life using SF-36.^[11,26,80]^ Twelve trials reported AEs.^[11,47,54,62,65,71,73,75,76,78,80,81]^ Finally, of 25 trials conducting follow-ups, 10 trials reported the pain intensity after a follow-up period,^[23,26,33,49,52,57–59,75,80]^,which varied from 180 minutes to 9 months by study. Most studies reported various outcomes measuring dysmenorrhea and related symptoms.

### 3.3 Risk of bias

All 60 studies mentioned randomization. Twenty trials used random number tables,^[23,24,26,33,38,43,44,49,51,52,54,57–61,65,67,71,81]^ 10 used a computer-generated sequence,^[11,48,73–80]^ 5 used central random method,^[28,29,37,40,41]^ and 1 used the draw method.^[32]^ Three trials used the order of joining the study,^[34,69,72]^ and the other studies did not report the details of randomization. Sixteen studies reported appropriate allocation concealment^[11,28–30,37,40,41,47,48,73–77,79,80]^ using computer programs, central telephone controls, sealed envelopes, or independent individual controls.

It is difficult to achieve blinding to both participants and practitioners for the characteristics of study design in acupuncture intervention, but 14 studies mentioned efforts to minimize performance bias,^[23,28,40,47,48,73–81]^ so we assessed the risk of bias as low. Twelve studies reported assessor blinding,^[23,28,40,48,73–80]^ but most of the others did not report the details.

**Figure 2:**
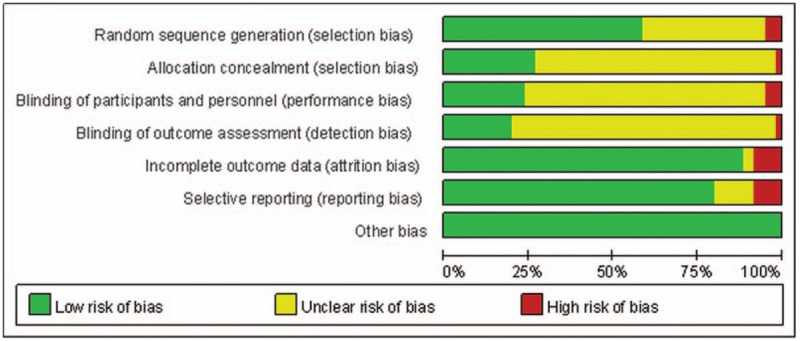
Risk of bias graph: review of the authors’ judgments regarding each risk of bias item presented as percentages across all included studies.

**Figure 3:**
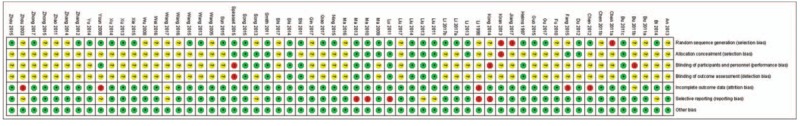
Risk of bias summary: review of the authors’ judgments regarding each risk of bias item in each included study.

Most of the studies had no missing data, performed intention-to-treat (ITT) analysis, or had similar numbers and reasons of drop-outs. However, the details of drop-outs and withdrawals were not reported in 6 studies,^[27,30,36,66,71,81]^ considered to be a high risk in reporting bias. Forty-nine studies reported all outcomes clearly as mentioned in protocol studies or methods^[11,23–25,27–34,37–39,41–46,48–65,67–72,75,78–80]^ and were assessed as a low risk of bias in selective reporting. Six studies reported the outcomes unclearly^[26,47,66,73,74,81]^ and were assessed as an unclear risk of bias, and 5 studies did not report all outcomes as planned^[35,36,40,76,77]^ and were assessed as a high risk of bias. There was a low risk of other sources of bias based on lack of clear evidence. As shown in Figures 2 and 3, most of the studies included in this meta-analysis achieved a low or unclear risk of bias of the quality assessment items.

### 3.4 Data synthesis

#### 3.4.1 Manual acupuncture

##### 3.4.1.1 MA versus no treatment

**Figure 4:**
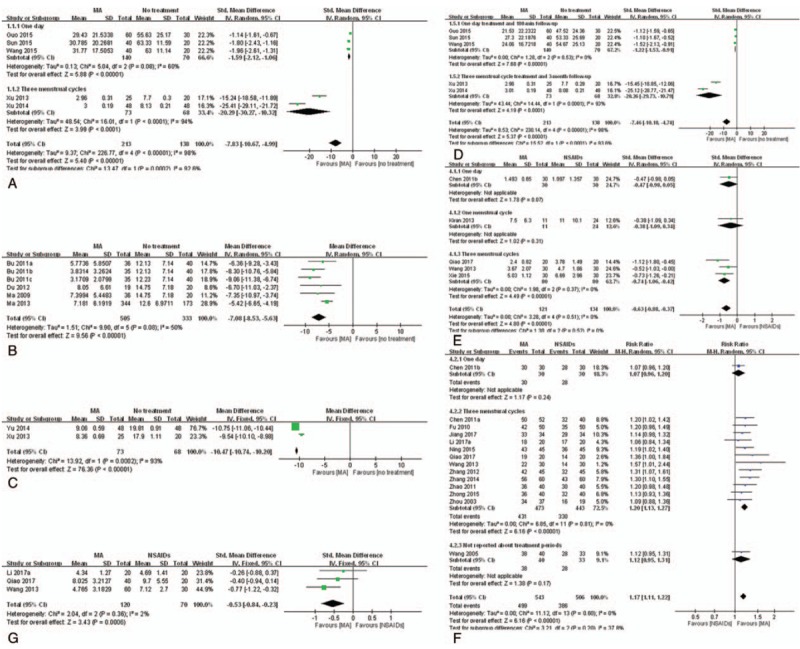
Meta-analysis of the studies evaluating the effects of MA on primary dysmenorrhea. (A) MA vs no treatment, outcome: VAS. (B) MA vs no treatment, outcome: CMSS for pain intensity. (C) MA vs no treatment, outcome: RSS. (D) MA vs no treatment, outcome: VAS after follow-up. (E) MA vs NSAIDs, outcome: VAS. (F) MA vs NSAIDs, outcome: TER. (G) MA vs NSAIDs, outcome: MSS. CMSS = Cox menstrual symptom scale, MA = manual acupuncture, NSAID = nonsteroidal anti-inflammatory drug, MSS = menstrual symptom score, RSS = Cox retrospective symptom scale, TER = total effective rate, VAS = visual analog scale.

*VAS.* Five studies^[33,49,52,58,59]^ were included in the meta-analysis to synthesize VAS data. As shown in Figure 4A, the pooled results showed serious heterogeneity (*I*^2^ = 98%). We conducted a subgroup analysis, and the pooled results showed that after treatment of 1 day, MA more effectively reduced primary dysmenorrhea than no treatment (n = 210, SMD = −1.59, 95% CI [−2.12, −1.06], *P* < .001, *I*^2^ = 60%).

*VRS.* One study^[60]^ reported that after the treatment of 3 menstrual cycles, MA more effectively reduced primary dysmenorrhea than no treatment (n = 96, MD = −2.04, 95% CI [−2.11, −1.97], *P* < .001).

*CMSS for pain intensity.* Six studies^[27–29,41,68,77]^ were included in the meta-analysis to synthesize CMSS for pain intensity data. As shown in Figure 4B, after treatment of 3 menstrual cycles, MA more effectively reduced pain than no treatment (n = 838, MD = −7.08, 95% CI [−8.53, −5.63], *P* < .001, *I*^2^ = 50%). Two studies^[25,37]^ reported the subscales of CMSS for pain intensity; 1 study^[25]^ reported the MA significantly reduced abdominal pain, and the other^[37]^ reported that the MA significantly reduced extra bed time.

*RSS.* Two studies^[58,60]^ were included for meta-analysis to synthesize RSS data. As shown in Figure 4C, after treatment of 3 menstrual cycles, MA more effectively reduced pain than no treatment (n = 141, MD = −10.47, 95% CI [−10.74, −10.20], *P* < .001, *I*^2^ = 93%).

*VAS after follow-up.* Five studies^[33,49,52,58,59]^ were included for meta-analysis to synthesize VAS after follow-up data. As shown in Figure 4D, the pooled results showed serious heterogeneity (*I*^2^ = 98%). We conducted a subgroup analysis, and after a 180-minute follow-up, MA was significantly more effective than no treatment (n = 210, SMD = −1.22, 95% CI [−1.53, −0.91], *P* < .001, *I*^2^ = 0%).

##### 3.4.1.2 MA versus placebo acupuncture

*Pain intensity.* Three studies reported pain scores,^[23,80,81]^ but data were unsuitable for pooling because the score systems of the studies were different from each other. One study^[23]^ reported that MA lowered monthly pain score after treatment of 3 menstrual cycles (n = 22, MD = −70.67, 95% CI [−126.52, −14.82], *P* = .01) and another study^[80]^ reported that MA lowered the pain score after treatment of 3 menstrual cycles without significant differences (n = 92, MD = −0.7, 95% CI [−1.8, 0.4], *P* = .21). The other study^[81]^ also reported that there were no significant differences between groups after treatment of 3 menstrual cycles (n = 47).

*Pain relief.* One study^[23]^ reported that after treatment of 3 menstrual cycles, MA provided a significant improvement in pain compared to placebo acupuncture (n = 22, RR = 2.50, 95% CI [1.12, 5.58], *P* < .05).

*SF-36.* One study^[80]^ reported that after treatment of 3 menstrual cycles, there was no significant difference in all SF-36 subscales or both component scores between the 2 groups (n = 92, bodily pain MD = −6.1, 95% CI [−15.1, 2.8], *P* = .18; General health MD = 5.1, 95% CI [−3.0, 13.2], *P* = .22; Vitality MD = 3.2, 95% CI [−5.1, 11.6], *P* = .44; Social function MD = 1.1, 95% CI [−8.0, 10.3], *P* = .81; Role emotional MD = 2.2, 95% CI [−12.7, 17.1], *P* = .77; Mental health MD = 6.0, 95% CI [−1.7, 13.6], *P* = .13; Overall Physical Component MD = −2.3, 95% CI [−5.9, 1.2], *P* = .19; Overall Mental Component MD = 3.5, 95% CI [−1.1, 8.1], *P* = .71).

*Pain intensity after follow-up.* Two studies^[23,80]^ reported this outcome. One study^[23]^ reported MA maintained pain reduction until 9 months after the completion of treatment (n = 22, MD = −64.90, 95% CI [−122.11, −7.69], *P* = .03). The other^[80]^ reported there were no significant differences between the groups after 3- and 9-month follow-up periods.

*AEs.* Two studies^[80,81]^ reported there were no AEs.

##### 3.4.1.3 MA versus oral medications

*Pain intensity.* Five studies^[44,51,57,70,72]^ comparing MA to NSAIDs reported VAS, and 1 study^[11]^ comparing MA to OCs reported a change in NRS. As shown in Figure 4E, the MA was significantly more effective at reducing pain than NSAIDs (n = 255, SMD = −0.63, 95% CI [−0.88, −0.37], *P* < .001, *I*^2^ = 0%). Meanwhile, OCs were more effective than MA after treatment of 3 menstrual cycles (n = 52, MD = 1.58, 95% CI [0.36, 2.80], *P* < .01).

*Pain relief.* Fourteen studies^[24,31,34,43,44,50,51,61–64,66,69,70]^ comparing MA to NSAIDs were included for meta-analysis to synthesize TER data. As shown in Figure 4F, MA provided significant pain relief compared to NSAIDs (n = 1,049, RR = 1.17, 95% CI [1.11, 1.22], *P* < .001, *I*^2^ = 0%). The funnel plot of those studies did not show asymmetry. One study^[67]^ reported pain relief as percentage using 6-Likert score, and also showed significant pain relief compared to NSAIDs (n = 84, RR = 2.97, 95% CI [1.75, 5.05], *P* < .01).

*MSS.* Three studies^[24,44,51]^ comparing MA to NSAIDs reported MSS. As shown in Figure 4G, MA was significantly more effective at improving menstrual symptoms than NSAIDs after treatment of 3 menstrual cycles (n = 190, SMD = −0.53, 95% CI [−0.84, −0.23], *P* < .001, *I*^2^ = 2%).

*SF-36.* One study^[11]^ reported that there was no significance difference between the 2 groups after treatment of 3 menstrual cycles (n = 52, MD = −1.82, 95% CI [−9.36, 5.72], *P* = .64).

*VAS after follow-up.* One study^[57]^ reported that after a 3-month follow-up, MA was significantly more effective than NSAIDs (n = 60, MD = −1.39, 95% CI [−2.65, −0.13], *P* < .05).

*AEs.* Two studies^[11,62]^ reported AEs. One study^[11]^ reported one case of regional discomfort or hemorrhage, 4 cases of headache or myalgia, and 1 case of fever in the MA group, which were all mild. Meanwhile, in the OCs group^[11]^, 9 cases of abnormal uterine bleeding, 5 cases of headache or myalgia, 3 cases of weight gain, 2 cases of nausea or vomiting, and 1 case of breast bleeding were reported; they were all already known AEs of OCs and were not severe. The other study^[62]^ reported 3 cases of elevated alanine transaminase (ALT), 2 cases of blurred vision, 3 cases of lumbar and leg pain, and 5 cases of others, which were predictable reactions, and soon disappeared.

#### 3.4.2 Electroacupuncture

##### 3.4.2.1 EA versus no treatment

*VAS.* Four studies^[47,73,76,78]^ reported that EA was significantly more effective at reducing pain than no treatment (n = 97, MD = −15.56, 95% CI [−22.16, −8.95], *P* < .001^[73]^; n = 26, MD = −23.19, 95% CI [−32.06, −14.33], *P* < .001^[76]^; n = 20, MD = −22.50, 95% CI [−31.70, −13.30], *P* < .005^[78]^; n = 97, *P* < .001; details of data not shown^[47]^). Data were unsuitable for pooling for means and SDs were not reported.

*VRS.* Two studies^[73,76]^ reported that there was no significant difference between the groups. Data were unsuitable for pooling because they reported the results only in graphs, which made it hard to extract raw data.

*RSS.* Three studies^[47,73,76]^ reported this outcome. Two studies^[73,76]^ showed there was no significant difference in RSS, but the other study^[47]^ showed that EA was significantly more effective than no treatment in RSS-COX2 (*P* < .05). Data were unsuitable for pooling because they reported the results only as graphs, which made it hard to extract raw data.

*AEs.* Four studies^[47,73,76,78]^ reported AEs, but 1study^[73]^ showed 1 case of dizziness after EA.

##### 3.4.2.2 EA versus placebo acupuncture

**Figure 5:**
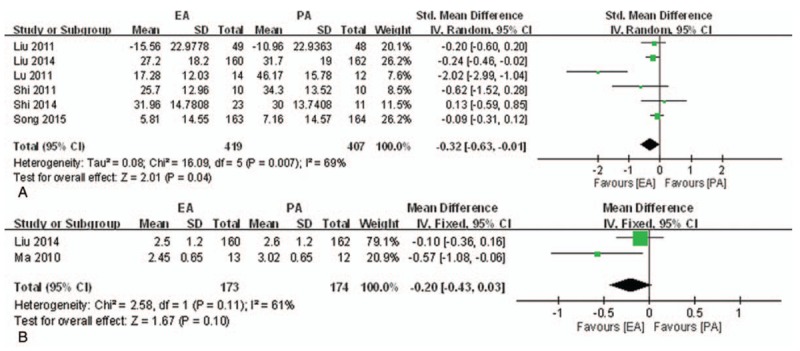
Meta-analysis of the studies evaluating the effects of EA on primary dysmenorrhea. (A) EA versus PA, outcome: VAS. (B) EA versus PA, outcome: VRS. (C) EA versus NSAIDs, outcome: TER. EA = electroacupuncture, IV = inverse variance, NSAIDs = nonsteroidal inflammatory drugs, NSAID = nonsteroidal anti-inflammatory drug, PA = placebo acupuncture, SD = standard deviations, TER = total effective rate, VAS = visual analog scale, VRS = seven-point verbal rating scale.

*VAS.* Nine studies^[40,47,48,73–76,78,79]^ reported this outcome; only 6 studies^[40,48,73,75,78,79]^ were included in the meta-analysis because the other 3 did not provide SDs. As shown in Figure 5A, the VAS of the EA group was significantly lower than placebo group (n = 826, SMD = −0.32, 95% CI [−0.63, −0.01], *P* = .04, *I*^2^ = 69%). Of the 3 studies excluded from meta-analysis^[47,74,76]^, 1 study^[74]^ reported that EA was significantly more effective in reducing pain than the placebo group in cold-dampness stagnation after one session of treatment (n = 487, MD = −8.2, 95% CI [−13.5, −2.9], *P* < .005); in other types, there was no significant difference. Another study^[76]^ showed the same result after treatment of 1 menstrual cycle (n = 25, MD = −20.78, 95% CI [−29.82, −11.73], *P* < .001). The other RCT^[47]^ reported that there was no significant difference between the groups after treatment of 1 menstrual cycle (n = 97, details of data not shown).

*VRS.* Three studies^[73,75,76]^ reported VRS, but only 2^[75,76]^ were included in the meta-analysis because the third did not provide SDs. As shown in Figure 5B, after treatment of 1 menstrual cycle, the VRS in the EA group was lower than the placebo group, but there was no significance (n = 347, MD = −0.20, 95% CI [−0.43, 0.03], *P* = .10, *I*^2^ = 61%). The other study^[73]^ also reported a change of VRS in the EA group that was lower than the placebo group, but there was no significance (n = 322, reduction from 3.94 to 3.08 vs reduction 3.72 to 3.02).

*RSS.* Four studies^[47,73,75,76]^ reported RSS, but the data were unsuitable for pooling due to insufficient reporting. One study^[73]^ reported there was no significant difference between the groups (RSS-COX1: reduction from 18.98 to 18.38 vs reduction from 19.44 to 20.20; RSS-COX2: reduction from 11.88 to 11.38 vs reduction from 10.22 to 10.51), and another study^[75]^ showed no significance after 3 cycles, either (n = 97, RSS-COX1 MD = −0.8, 95% CI [−2.2, 0.7], *P* = .28; RSS-COX2 MD = −0.5, 95% CI [−1.4, 0.4], *P* = .28). The other 2 studies^[47,76]^ also showed no significance in RSS (data not shown).

*VAS after follow-up.* One study^[75]^ reported that after one-cycle follow-up, there were no significance differences between EA and PA groups (n = 322, MD = −4.40, 95% CI [−9.55, 0.75], *P* = .09).

*AEs.* Five studies^[47,73,75,76,78]^ reported AEs. Three studies^[47,76,78]^ reported there were no AEs, and one^[73]^ of the other studies reported one case of dizziness after EA. The other study^[75]^ reported one case of minimal bleeding in the EA group, and one case of minimal bleeding and one case of pain after insertion in the placebo group.

##### 3.4.2.3 EA versus NSAIDs

*VAS.* One study^[55]^ reported that after treatment of 3 menstrual cycles, EA was significantly effective at reducing pain than NSAIDs (n = 60, MD = −1.40, 95% CI [−2.21, −0.59], *P* < .01).

*Pain relief.* Two studies^[30,55]^ reported TER, but as shown in Figure 5C, the pooled results showed no significant differences between 2 groups (n = 140, RR = 1.80, 95% CI [0.99, 1.18], *P* = .09, *I*^2^ = 0%).

#### 3.4.3 Auricular acupuncture

##### 3.4.3.1 AA versus NSAIDs

*VAS.* One study^[38]^ reported that after treatment of 3 menstrual cycles, there were no significant differences between the 2 groups (n = 70, MD = −0.20, 95% CI [−0.90, −0.50], *P* = .58).

#### 3.4.4 Warm acupuncture

##### 3.4.4.1 WA versus NSAIDs

**Figure 6:**
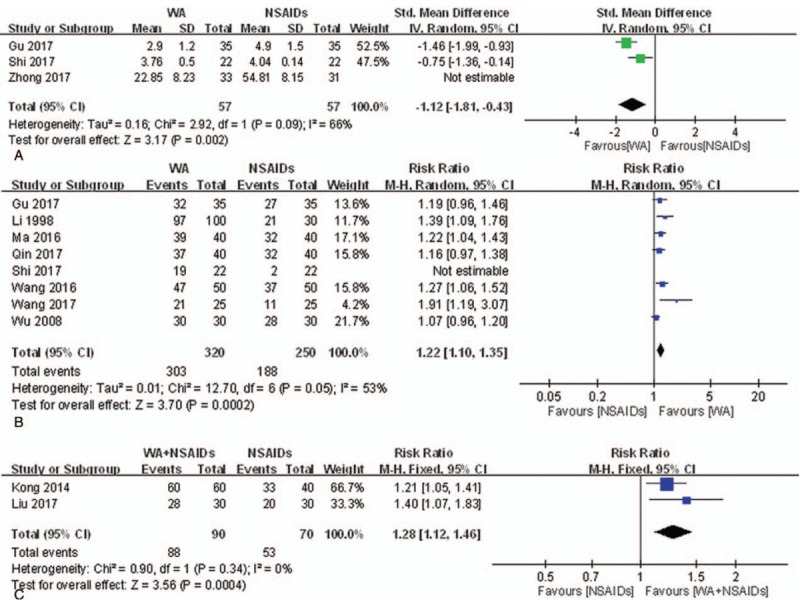
Meta-analysis of the studies evaluating the effects of WA on primary dysmenorrhea. (A) WA vs NSAIDs, outcome: VAS. (B) WA vs NSAIDs, outcome: TER. (C) WA plus NSAIDs vs NSAIDs, outcome: TER. CI = confidence interval, IV = inverse variance, NSAIDs = nonsteroidal anti-inflammatory drugs, SD = standard deviations, TER = total effective rate, VAS = visual analog scale, WA = warm acupuncture.

*VAS.* Three studies^[32,46,65]^ were included in the meta-analysis to synthesize VAS data. A meta-analysis of the 3 studies involving 178 participants was implemented, but the results showed serious heterogeneity (*I*^2^ = 94%). We conducted a sensitivity analysis by excluding the trial^[65]^ with effect sizes largely different from the others. Statistical heterogeneity was reduced after exclusion. As shown in Figure 6A, with 2 remaining studies, the VAS of the WA group was significantly lower than NSAIDs group (n = 114, SMD = −1.12, 95% CI [−1.81, −0.43], *P* = .002, *I*^2^ = 66%).

*TER.* Eight studies^[32,36,42,45,46,53,54,56]^ were included in the meta-analysis to synthesize TER data. A meta-analysis of the 3 studies involving 614 participants was implemented, but the results showed serious heterogeneity (*I*^2^ = 77%). We conducted a sensitivity analysis by excluding the trial^[46]^ with effect sizes largely different from the others. As shown in Figure 6B, with 6 remaining studies, the WA provided significant pain relief compared to NSAIDs after the treatment of 3 menstrual cycles (n = 570, RR = 1.22, 95% CI [1.10, 1.35], *P* < .001, *I*^2^ = 53%).

*CMSS for pain intensity.* One study^[32]^ reported that after the treatment of 3 menstrual cycles, WA more effectively reduced primary dysmenorrhea than NSAIDs (n = 70, MD = −8.00, 95% CI [−9.54, −6.46], *P* < .001).

*MSS.* One study^[45]^ reported that there were no significant differences between WA and NSAIDs groups (n = 80, MD = −1.09, 95% CI [−2.65, −0.47], *P* = .17).

*AEs.* Two studies^[54,65]^ reported AEs. One study^[54]^ reported 5 cases of nausea, vomiting, and fever in the NSAIDs group, and the other study^[65]^ reported there were no AEs.

##### 3.4.4.2 WA plus NSAIDs versus NSAIDs

*TER.* Two studies^[35,39]^ reported that WA adding on NSAIDs provided significant pain relief compared to only NSAIDs after treatment of 3 menstrual cycles (n = 160, RR = 1.28, 95% CI [1.12, 1.46], *P* < .001, *I*^2^ = 0%).

#### 3.4.5 Catgut embedding therapy

##### 3.4.5.1 CET versus NSAIDs

*VAS.* One study^[26]^ reported that CET was significantly more effective than NSAIDs after treatment of 3 menstrual cycles (n = 70, *t* = −2.70, *P* < .01).

**Figure 7:**
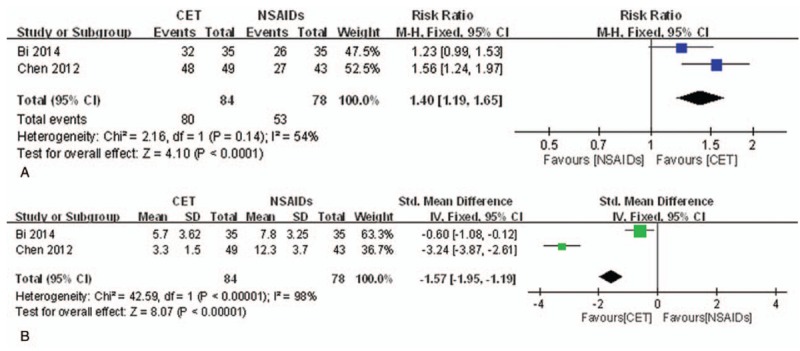
Meta-analysis of the studies evaluating the effects of CET on primary dysmenorrhea. (A) CET vs NSAIDs, outcome: TER. (B) CET vs NSAIDs, outcome: MSS. CET = catgut embedding therapy, CI = confidence interval, IV = inverse variance, MSS = menstrual symptom score, NSAIDs = nonsteroidal anti-inflammatory drugs, SD = standard deviations, TER = total effective rate.

*TER.* Two studies^[26,71]^ reported that CET provided significant pain relief compared to NSAIDs after treatment of 3 menstrual cycles as shown in Figure 7A (n = 162, RR = 1.40, 95% CI [1.19, 1.65], *P* < .001, *I*^2^ = 54%).

*MSS.* Two studies^[26,71]^ reported that CET effectively reduced menstrual symptoms compared to NSAIDs after treatment of 3 menstrual cycles with serious heterogeneity as shown in Figure 7B (n = 162, SMD = −1.57, 95% CI [−1.95, −1.19], *P* < .001, *I*^2^ = 98%).

*SF-36.* One study^[26]^ reported that SF-36 in the CET group was significantly higher than the NSAIDs group after treatment of 3 menstrual cycles (n = 70, t = 2.535, *P* < .05).

*VAS after follow-up.* One study^[26]^ reported that after a 3-month follow-up, CET was significantly more effective than NSAIDs (n = 70, t = −4.72, *P* < .01).

*AEs.* One study^[71]^ reported 6 cases of gastrointestinal discomforts, headache, dizziness, and insomnia in the NSAIDs group.

## 4 Discussion

### 4.1 Summary of the main results

This systematic review was aimed to summarize and evaluate acupuncture treatment to reduce menstrual pain and its associated symptoms. As a result, we suggest that acupuncture might have beneficial effects for improvement of dysmenorrhea and remain efficacious after short-term follow-up.

We conducted comparisons separately according to the characteristics of interventions and controls. MA was significantly more effective than no treatment, and NSAIDs for reduction of menstrual pain and its associated symptoms, and remained effective after a short-term follow-up compared to no treatment and NSAIDs. The MA-induced analgesic effect could be explained by C-fiber involvement during the practitioners’ manipulation for the *de-qi* response.^[85]^ However, no significant difference was observed between MA and placebo acupuncture or between MA and OCs. It was difficult to determine the superior effect of OCs compared to MA because there was only one relevant study.^[11]^

The results showed that EA was significantly more effective at reducing menstrual pain than no treatment,^[47,73,76,78]^ placebo acupuncture,^[40,48,73–76,78,79]^ but not effective at improving its associated symptoms.^[47,73,75,76]^ The results comparing with NSAIDs were insufficient to determine the efficacy of EA. The mechanism of EA-induced analgesia could be explained by inducing the release of endorphins^[86,87]^ and the decrease of the pulsatility index in the uterine arteries,^[88]^ which might be related to primary dysmenorrhea.^[1]^ The reason that there was no difference between MA and placebo acupuncture and the relatively small difference between EA and placebo acupuncture was thought to be that placebo acupuncture also had positive effects. Several factors might explain the positive effects. First, some participants receiving placebo acupuncture may want pain relief, and it may affect the outcome psychologically.^[89]^ Second, placebo acupuncture may stimulate cutaneous touch receptors and/or skin nociceptors and modulate the activity in the brain areas associated with pain management.^[90]^

WA was significantly more effective at reducing menstrual pain than NSAIDs, but the efficacy for the associated symptoms was inconclusive due to the small sample size. The results showed WA with NSAIDs might also relieve menstrual pain compared to NSAIDs alone. WA increases the circulation of *qi* and blood through the needle body during thermal heating. It provides analgesic effects by stimulating nerve transfer and relaxing uterine muscle spasms.^[91]^

CET might also be effective for primary dysmenorrhea. CET is a therapeutic modality based on acupuncture theory and continuous stimulation of acupoints with embedded thread, and its continuous stimulation prolongs the effects of acupuncture. In addition, the embedded thread gradually liquefies and is absorbed, and stimulates the points physically and chemically.^[26]^ With this mechanism, CET might be considered to demonstrate analgesic effects and maintain the effects for short-term follow-up.

Severe AEs of acupuncture were not observed. Thirteen of the 60 studies reported AEs of acupuncture. Most of the reported AEs were regional pain or discomfort, hematoma, and dizziness. Those mentioned were mild, similar to previously known AEs.^[92]^

The applicability of acupuncture to primary dysmenorrhea in other settings is unclear. Fifty-seven of the trials were conducted in Asian countries: 55 in China, 1 in Thailand, and 1 in South Korea. The acupuncture practitioners might have different treatment skills according to the nations in which they were trained, and the participants might have different preconceptions and familiarity with acupuncture according their cultures.^[89]^ In addition, the variability of the details of interventions and controls could make applicability unclear.

### 4.2 Strengths and limitations of this review

Six SRs which evaluate the efficacy of acupuncture on primary dysmenorrhea have previously been conducted,^[14–19]^ and 2 of them were published in 2016^[17]^ and 2017^[19]^, respectively. However, there were some differences between these 2 SRs and our review. They may arise from the different search strategies, inclusion criteria, and analysis methods. In particular, the Cochrane review^[17]^ analyzed 42 studies, just separating the treatment types into acupuncture and acupressure. Liu et al's^[19]^ review analyzed 23 studies with similar strategies to our review, did not include 10 trials newly published in 2017, and did not include other modalities of acupuncture such as WA or CET, frequently used in clinical fields. Our review included all types of acupuncture that stimulate acupoints by penetrating the skin, including CET, and synthesized data separately according to the characteristics of the interventions and controls.

Our study had some limitations, and those results mentioned above should be interpreted with caution. One was that most of the included trials achieved a low or unclear risk of bias. The unclear judgements appeared mostly in the domains of allocation concealment and blinding of participants/practitioners/outcome assessors, because the details were not described. The blinding of participants is critical for subjective outcomes such as pain,^[93]^ but blinding of both participants and practitioners was difficult due to the characteristics of acupuncture intervention. The other limitation was that there was substantial heterogeneity among the pooled trials. We tried to reduce the heterogeneity by synthesizing the data separately depending on the characteristics of the interventions and controls, subgroup analysis, and sensitivity analysis, but the unresolved heterogeneity in some cases still existed. We considered this heterogeneity derived from the small sample sizes in some outcomes and the methodological variations among the included studies. The methods of interventions varied in the frequency, duration of each session, selection of acupoints, and *de-qi* methods. The variations of controls also appeared in different components of NSAIDs. These variations could influence the results of the trials, and were considered to cause unresolved heterogeneity.

### 4.3 Implications of this review for practice and research

To provide convincing evidence of the efficacy of acupuncture for primary dysmenorrhea, future RCTs should adhere to rigorous standards assessing the risk of bias, such as conducting randomization allocation concealment and trying to avoid performance bias. In addition, those trials should be reported as STRICTA guidelines^[84]^ to clear the specific method of each intervention.

## 5 Conclusions

The results of this study suggest that acupuncture might reduce menstrual pain and associated symptoms more effectively compared with no treatment or NSAIDs, and the efficacy could be maintained during a short-term follow-up period. However, the efficacy of acupuncture compared to a placebo was not convincing. The safety of acupuncture appeared because a few mild AEs were reported. Our suggestions had limitations because the quality of the included RCTs was low, and methodological restriction existed in this study. More rigorously designed trials are required to confirm our findings.

## Author contributions

**Conceptualization:** Hye Lin Woo, Hae Ri Ji, Yeon Kyoung Pak, Jin Moo Lee, Kyoung Sun Park.

**Data curation:** Hye Lin Woo, Yeon Kyoung Pak, Hojung Lee, Su Jeong Heo.

**Formal analysis:** Hye Lin Woo.

**Funding acquisition:** Jin Moo Lee.

**Methodology:** Hye Lin Woo, Hae Ri Ji, Yeon Kyoung Pak, Hojung Lee, Su Jeong Heo, Kyoung Sun Park.

**Project administration:** Jin Moo Lee, Kyoung Sun Park.

**Writing – original draft:** Hye Lin Woo.

**Writing – review & editing:** Kyoung Sun Park.

## Supplementary Material

Supplemental Digital Content
