# Supplementary material for: The efficacy and safety of acupuncture in women with primary dysmenorrhea: A systematic review and meta-analysis
Source: Medicine (Baltimore). 2018 Jun 18;97(23):e11007. doi: 10.1097/MD.0000000000011007 (PMC5999465; doi:10.1097/MD.0000000000011007)
Supplement: Supplemental Digital Content [file medi-97-e11007-s001.docx]

**Supplemental Search Terms List**

*MEDLINE*

#1. dysmenorrhea[mh]

#2. dysmenorrh*[tiab]

#3. menstrual pain*[tiab]

#4. painful menstruation*[tiab]

#5. primary dysmenorrh*[tiab]

#6. #1 OR #2 OR #3 OR #4 OR #5

#7. Acupuncture Therapy[mh]

#8. Acupuncture Analgesia[mh]

#9. Acupuncture, Ear[mh]

#10. Electro-acupuncture[mh]

#11. Meridians[mh]

#12. Acupuncture Points[mh]

#13. Acupressure[mh]

#14. Acup*[tiab]

#15. acupuncture[tiab]

#16. acupuncture analgesia*[tiab]

#17. acupuncture point*[tiab]

#18. acupuncture therap*[tiab]

#19. Electro-acupuncture*[tiab]

#20. electro acup*[tiab]

#21. electro-acupuncture*[tiab]

#22. akupuncture[tiab]

#23. acupoint*[tiab]

#24. needl*[tiab]

#25. meridian*[tiab]

#26. auricular acup*[tiab]

#27. auricular point*[tiab]

#28. ear acup*[tiab]

#29. sham acup*[tiab]

#30. sham point*[tiab]

#31. sham needl*[tiab]

#32. #7 OR #8 OR #9 OR #10 OR #11 OR #12 OR #13 OR #14 OR #15 OR #16 OR #17 OR #19 OR #20 OR #21 OR #22 OR #23 OR #24 OR #25 OR #26 OR #27 OR #28 OR #29 OR #30 OR #31

#33. #6 AND #32

*EMBASE*

#1. "dysmenorrhea*".ab,ti.

#2. "menstrual pain*".ab,ti.

#3. "painful menstruation*".ab,ti.

#4. "primary dysmenorrh*".ab,ti.

#5. #1 or #2 or #3 or #4

#6. "acup*".ab,ti.

#7. acupuncture.ab,ti.

#8. acupuncture analgesia.ab,ti.

#9. acupuncture points.ab,ti.

#10. acupuncture therapy.ab,ti.

#11. "electro acup*".ab,ti.

#12. Electro-acupuncture.ab,ti.

#13. akupuncture.ab,ti.

#14. acupoint.ab,ti.

#15. "needl*".ab,ti.

#16. needling.ab,ti.

#17. "meridian*".ab,ti.

#18. "ear acup*".ab,ti.

#19. "acupress*".ab,ti.

#20. "sham needl*".ab,ti.

#21. "sham acup*".ab,ti.

#22. "sham point*".ab,ti.

#23. #6 or #7 or #8 or #9 or #10 or #11 or #12 or #13 or #14 or #15 or #16 or #17 or #18 or 19 or #20 or #21 or #22

#24. #5 and #23

*CENTRAL*

#1. MeSH descriptor:[Dysmenorrhea]explode all trees

#2. dysmenorrhea*:ab,ti

#3. menstrual pain*:ab,ti

#4. painful menstruation*:ab,ti

#5. primary dysmenorrh*:ab,ti

#6. #1 or #2 or #3 or #4 or #5

#7. MeSH descriptor:[acupuncture]explode all trees

#8. MeSH descriptor:[acupuncture analgesia]explode all trees

#9. MeSH descriptor:[acupuncture points]explode all trees

#10. MeSH descriptor:[acupuncture therapy]explode all trees

#11. MeSH descriptor:[Electro-acupuncture]explode all trees

#12. acupuncture:ab,ti

#13. akupuncture:ab,ti

#14. acupoint*:ab,ti

#15. electro acupuncture:ab,ti

#16. needl*:ab,ti

#17. needling:ab,ti

#18. meridian*:ab,ti

#19. electro acup*:ab,ti

#20. ear acup*:ab,ti

#21. acupress*:ab,ti

#22. sham needl*:ab,ti

#23. sham acup*:ab,ti

#24. sham point*:ab,ti

#25. #7 or #8 or #9 or #10 or #11 or #12 or #13 or #14 or #15 or #16 or #17 or #18 or 19 or #20 or #21 or #22 or #23 or #24

#26. #6 and #25
